# Early radiographic and functional outcomes of a cancellous titanium-coated tibial component for total knee arthroplasty

**DOI:** 10.1007/s12306-015-0382-z

**Published:** 2015-09-26

**Authors:** D. D. Waddell, K. Sedacki, Y. Yang, D. A. Fitch

**Affiliations:** Orthopedic Specialists of Louisiana, Shreveport, LA USA; MicroPort Orthopedics Inc., 5677 Airline Rd., Arlington, TN 38002 USA

**Keywords:** Total knee arthroplasty, Total knee replacement, BIOFOAM, Cementless fixation, ADVANCE

## Abstract

**Background:**

Various surface coatings have been developed over the past decades to enhance fixation of cementless total knee arthroplasty (TKA). BIOFOAM^®^ (MicroPort Orthopedics Inc., Arlington, TN, USA) is a novel cancellous titanium surface coating intended to increase both initial and long-term fixation. The purpose of this study was to investigate the early functional and radiographic outcomes of this coating used in a TKA application.

**Materials and methods:**

One hundred and four (104) primary TKAs in 85 subjects using BIOFOAM-coated tibial components were prospectively enrolled at four centers. Subjects were evaluated using Knee Society Scores and radiographic analysis at a minimum follow-up of 24 months.

**Results:**

Knee Society Scores and flexion were all significantly improved at final follow-up compared to baseline. Radiographic analyses were satisfactory, with no progressive radiolucencies and only a single subject presenting with a radiolucency surrounding a tibial component. There were two revisions in the cohort: one for instability following a ruptured lateral collateral ligament and one for recurrent tibial insert dislocation.

**Conclusions:**

This is the first study to report clinical outcomes associated with the BIOFOAM coating used in a cementless TKA application. Early functional scores and radiographic analyses are promising, but further investigations are needed to confirm long-term clinical success with these components.

## Introduction

**Fig. 1:**
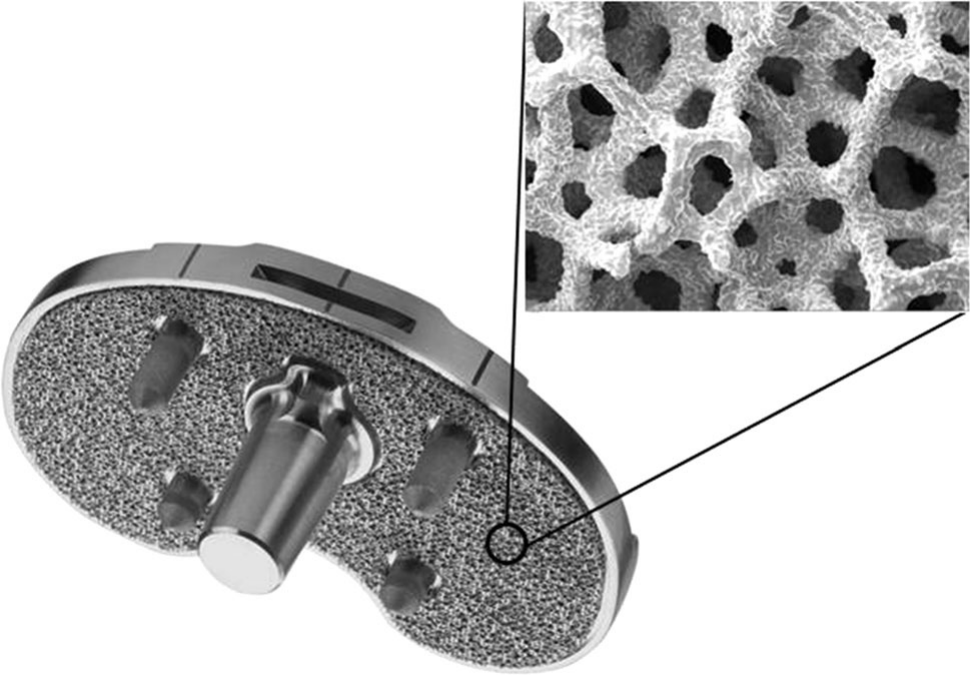
ADVANCE^®^ BIOFOAM^®^ tibial component is shown. The 100× magnification displays the roughened and porous surface of BIOFOAM. These components feature a Morse taper that allows for the attachment of various stem options

The ADVANCE^®^ Medial-Pivot System (MicroPort Orthopedics Inc., Arlington, TN, USA) was first introduced in 1998 by Wright Medical Technology, Inc. The cemented version of this total knee arthroplasty (TKA) system has been shown to have a long, satisfactory clinical history [1–6]. The system was later expanded to include titanium porous bead-coated components for cementless or hybrid fixation, and eventually a tibial component featuring a cancellous titanium coating commercially called BIOFOAM^®^ (Fig. 1).

BIOFOAM is a porous reticulated titanium material developed for load-bearing orthopedic applications and features a compressive modulus similar to that of native bone [7]. This is significant as differences in mechanical properties between native bone and implants can lead to stress shielding resulting in radiolucencies and decreased bone mineral density. A recent study found evidence of this occurring in TKAs performed using both cemented and titanium porous bead cementless fixation [8]. BIOFOAM also has other material properties (e.g., increased porosity and coefficient of friction) that make it attractive for the use in cementless TKA, but clinical outcomes have not yet been reported for this application. The objective of this preliminary study was to evaluate the short-term radiographic and functional outcomes for subjects implanted with BIOFOAM-coated tibial components used for cementless TKA.

## Materials and methods

One hundred and four (104) TKAs were performed in 85 subjects using the BIOFOAM-coated tibial components. Implantations were performed at four surgical centers by four surgeons. Institutional review board approval was obtained at each site prior to the enrollment of any subjects. Subjects were included if they required a TKA, were considered skeletally mature, and were considered to have a life expectancy to exceed 2 years. Subjects were excluded if they had a body mass index (BMI) greater than 45, an active infection, or were pregnant. All tibial components were implanted with cementless fixation, and femoral component fixation was at the discretion of the implanting surgeon. The patella was resurfaced in 26.0 % of TKAs, all using cemented patellar components. TKAs were performed using the subvastus (70.2 %), medial parapatellar (28.8 %), or midvastus (0.9 %) surgical techniques. The mean tourniquet time was 42.9 min (range 23.0–125.0 min). Demographics for included subjects are listed in Table 1.

**Table 1:** Demographics for included subjects

Age	64 years (range 43.0–87.0)
Male/female	44 (42.3 %)/60 (57.7 %)
BMI	32.0 kg/m^2^ (range 22.8–44.7 kg/m^2^)
Primary indications	
Degenerative osteoarthritis	102 (98.1 %)
Avascular necrosis	2 (1.9 %)

**Fig. 2:**
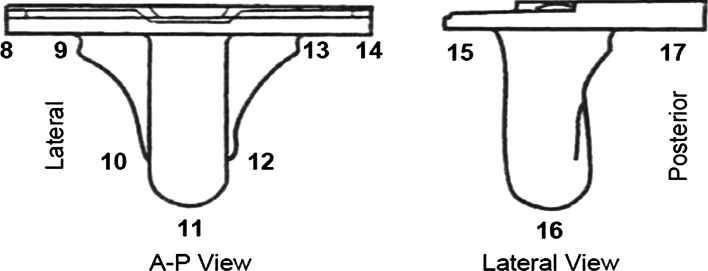
Tibial component radiographic zones used to evaluate radiolucencies

Subjects were evaluated preoperatively, and at 6, 12, and 24 months postoperatively. Knee Society Scores (total and function) and maximum flexion were used to assess functional outcomes [9]. Anterior–posterior (AP), lateral, and sunrise radiographs were also collected at each postoperative interval. Radiographs were reviewed for the number and size of radiolucencies in the 17 zones shown in Fig. 2 and for the evaluation of patella tracking.

### Statistical analysis

Knee Society Scores and maximum flexion at each interval were reported using mean and standard deviation values. These values were compared to baseline using a paired *t* test (*p* < 0.05). Radiolucencies were reported as the number and mean size occurring in each zone. All statistical analyses were performed using SAS version 9.1.3 (SAS Institute Inc., Cary, NC).

## Results

**Fig. 3:**
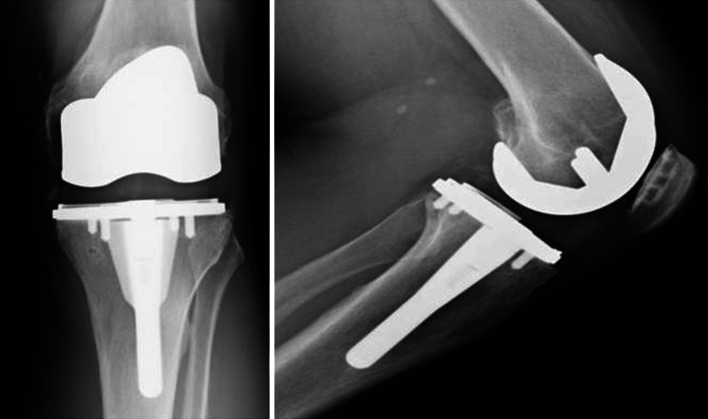
Representative anterior–posterior and lateral radiographs of a well-fixed BIOFOAM-coated tibial component

At each follow-up interval, Knee Society Scores (total and function) were significantly improved from baseline (Table 2). Flexion was significantly improved at 12 and 24 months. Representative radiographs of a well-fixed BIOFOAM tibial component are shown in Fig. 3. One subject experienced a 1-mm radiolucency in Zone 14 at 24 months. No other subjects presented with tibial zone radiolucencies. None of the tibial radiolucencies were progressive, and there were no signs of loosening or subsidence in either group. Six (6) subjects had radiolucencies in femoral zones at 24 months: 4 subjects with a radiolucency in Zone 1 (average size of 1.25 mm); 1 subject with a 1-mm radiolucency in Zone 5; and 1 subject with 1-mm radiolucencies in Zones 6 and 7, respectively. Radiographic analysis of the patella showed 94.0 % (100 % of resurfaced patellae and 91.7 % of non-resurfaced patellae) were tracking normally at 24 months. Of the five patellae not tracking normally, three were tilted and two were subluxed.

**Table 2:** Mean Knee Society Scores and range of motion values at each follow-up interval

	Mean ± Std. dev.	*p* value
Knee Society Scores		
Preoperative	53.6 ± 15.2	–
6 months	87.2 ± 14.3*	<0.001
12 months	90.0 ± 10.3*	<0.001
24 months	93.3 ± 10.0*	<0.001
Knee Society Function Scores		
Preoperative	52.6 ± 15.2	–
6 months	83.9 ± 17.0*	<0.001
12 months	87.0 ± 16.3*	<0.001
24 months	87.5 ± 18.5*	<0.001
Maximum flexion (°)		
Preoperative	110.3 ± 15.1	–
6 months	111.5 ± 13.1	0.05
12 months	114.8 ± 11.4*	0.008
24 months	116.8 ± 11.4*	<0.001

* Represents a statistically significant difference from preoperative values (*p* = 0.05)

There were two revisions in the cohort. One TKA was revised at 2 months due to instability following a ruptured lateral collateral ligament (LCL). Another was revised at 16 months for recurrent dislocation of the tibial insert. This subject also experienced several complications prior to revision including: pain at 5.7 months, displacement and revision of the tibial insert at 5.8 months, and a stress fracture of the fifth metatarsal at 7 months. Other complications for the cohort included: one hematoma, one case of unexplained pain, one foot drop, and one decreased range of motion treated with manipulation under anesthesia. There were no intraoperative complications.

## Discussion

Aseptic loosening remains one of the most common indications for revision TKA, accounting for over 30 % of these procedures performed in the UK during 2012 [2]. TKA with cemented fixation continues to be popular because it can reduce the risk of early loosening, but it does have some potential drawbacks compared to cementless fixation including increased operating/tourniquet time [10] and increased risk of thromboembolism [11]. Because aseptic loosening persists as a failure mode and to gain the benefits of cementless fixation, various surface finishes and component designs have been developed over the past several decades. One such technology is the BIOFOAM-coated tibial component.

BIOFOAM has a porosity of up to 70 %, which is significantly higher than that measured in titanium porous bead coatings [12]. A previous animal study compared bone ingrowth present in cylinders coated with either BIOFOAM- or titanium-sintered beads implanted in the diaphyses of nine dogs [7]. At 12-week follow-up, histological analysis showed there was more bone present in the BIOFOAM-coated specimens than there was space available for bone ingrowth in the porous bead specimens. BIOFOAM has a coefficient of friction that is over twice that of titanium porous bead coatings and nearly double that of titanium plasma spray coatings, which suggests that BIOFOAM is capable of providing increased resistance to motion prior to bone ingrowth when compared to other surface coatings [12]. While these characteristics make the material ideal for enhancing both short- and long-term implant fixation, clinical outcomes for these components have not been previously reported. In the current study, there were no complications or revisions related to migration or aseptic loosening, potentially supporting these described theoretical advantages.

BIOFOAM also has a compressive modulus similar to that of native bone, which may lead to more natural load transfers and reductions in stress shielding as previously described [7]. In the current study, only a single subject (1.2 %) presented with a radiolucency surrounding a BIOFOAM tibial component. Mean postoperative Knee Society Scores were excellent at final follow-up and similar to values previously reported in the literature for TKA. Revisions and complications were rare, and there were no failures attributable to the use of the BIOFOAM material or that have not been reported for other implant designs and coatings.

### Limitations

The main limitations of the current study are the relatively small sample size and the duration of follow-up. None of the observed radiolucencies progressed during early study visits, but it is possible they could during later follow-up. There is also the potential for the development of new radiolucencies over time.

## Conclusions

In conclusion, this preliminary study shows promising evidence for the use of BIOFOAM-coated tibial components in cementless TKA. Only a single subject experienced a radiolucency surrounding a tibial component, and functional outcomes were excellent. Further investigations are needed to confirm whether these positive early results continue in the long-term and whether outcomes for these components differ from other fixation methods.
